# HINT2 downregulation promotes colorectal carcinoma migration and metastasis

**DOI:** 10.18632/oncotarget.14587

**Published:** 2017-01-10

**Authors:** Weihua Li, Shaoxin Cai, Le Wang, Changshun Yang, Biaohuan Zhou, Huan Wang

**Affiliations:** ^1^ Department of Surgical Oncology, Fujian Provincial Clinical College, Fujian Medical University, Fuzhou 350001, China

**Keywords:** colorectal cancer, HINT2, epithelial–mesenchymal transition, HIF-2α, ZEB1

## Abstract

Histidine triad nucleotide-binding 2 (HINT2), a member of the histidine triad proteins family, sensitizes cells to apoptosis in hepatocellular carcinoma. Here, we showed that HINT2 expression is lower in primary colorectal cancer (CRC) and metastasis tissues than in normal colorectal tissues, and that HINT2 abundance is inversely correlated with CRC tumor stage. Treating CRC cells with 5-aza-2′-deoxycytidine, a demethylating agent, upregulated HINT2, suggesting HINT2 downregulation is caused by methylation of the gene promoter. HINT2 downregulation increased tumor migration and invasion *in vitro*, promoted CRC cell metastasis *in vivo*, and increased expression of epithelial-to-mesenchymal transition (EMT) markers. Furthermore, HINT2 downregulation depended on hypoxia inducible factor (HIF)-2α-mediated transcriptional activation of zinc finger E-box-binding homeobox 1 (ZEB1). These results suggest that HINT2 downregulation promotes HIF-2α expression, which induces EMT and enhances CRC cell migration and invasion. HINT2 may thus a useful clinical indicator of CRC progression and metastasis risk.

## INTRODUCTION

Colorectal cancer (CRC) is one of the most common malignant cancers, and approximately 50–60% of patients present with metastases at initial diagnosis [1–4]. Because advanced metastatic CRC remains largely incurable, there is an urgent need to elucidate the molecular mechanism underlying this aggressive cancer.

Histidine triad nucleotide-binding 2 (HINT2) is a member of the histidine triad superfamily that performs a range of biological functions. HINT2 is localized exclusively in the mitochondrial matrix and is a tumor suppressor in human hepatocellular carcinoma [5]. HINT2 also regulates lipid metabolism, glucose homeostasis, and mitochondrial respiration [6]. Mitochondria not only play a role in co-opting the regulatory pathways that inactivate anoikis, but also regulate metastatic cell survival [7–9]. Furthermore, quantitative real-time polymerase chain reaction (qRT-PCR) results revealed *HINT2* downregulation in colon carcinoma as compared to normal tissue [10]. Thus, HINT2 appears to be associated with CRC progression.

In the past decade, the epithelial–mesenchymal transition (EMT) has been increasingly recognized as promoting cancer cell invasion and metastasis [11, 12]. During EMT, a polarized epithelial cell undergoes multiple biochemical changes and acquires a mesenchymal cell phenotype, including loss of polarization, decreased cell-cell junctions, and increased motility [13]. After EMT, cells lose expression of the epithelial marker, E-cadherin (also known as CDH1), and begin expressing mesenchymal markers, such as vimentin, N-cadherin (also known as CDH2), Snail family zinc finger 1 (SNAI1), SNAIL2, Twist family bHLH transcription factor 1 (TWIST1), TWIST2, zinc finger E-box–binding homeobox 1 (ZEB1), and ZEB2 [14–19].

The present study analyzed the association of HINT2 with CRC metastasis. We found that downregulating HINT2 induces EMT in CRC cells and promotes CRC migration and metastasis *in vitro* and *in vivo*, respectively. HINT2 inhibits CRC cell invasion and migration by inducing EMT through ZEB1-mediated CDH1 inhibition via hypoxia inducible factor (HIF)-2α. ZEB1 knockdown via siRNA rescued the effects of HINT2 downregulation in CRC cells.

## RESULTS

### HINT2 expression in CRC tissue and cell lines

**Figure 1:**
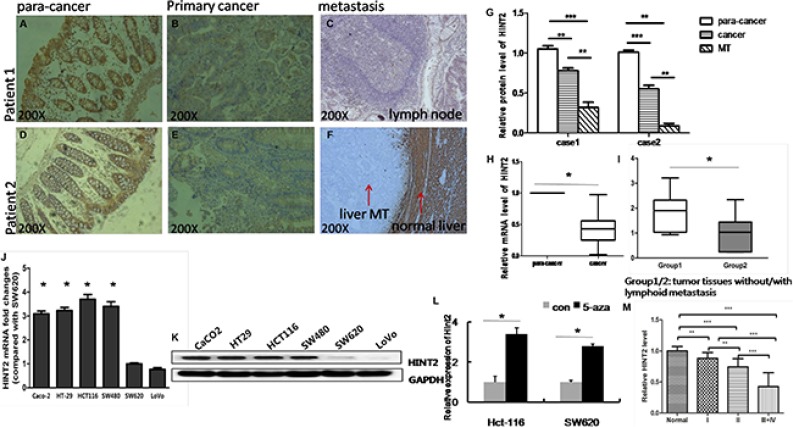
Endogenous HINT2 expression in CRC tissues and cell lines IHC staining of HINT2 in normal para-cancer (**A** and **D**), primary cancer (**B** and **E**), and metastasis tissues (**C**: lymph node MT, **F**: liver MT), and quantification (**G**) of the above. Arrows indicate liver metastasis and normal liver tissue, original magnification, ×200. (**H**) RT-qPCR analysis of HINT2 expression; data represent mean ± SD, *n* = 46 per group, *P < 0.05 H. RT-qPCR analysis of HINT2 expression; data represent mean ± SD, group 1 represents tissue without metastasis (*n* = 20), and group 2 represents tissue with metastasis (*n* = 26), *P < 0.05 (I). HINT2was detected in CRC cell lines via real-time PCR (**J**) and western blotting (**K**) Bar graph shows SW620 cell-normalized HINT2 expression in CRC cell lines. Hint2 was detected in Lovo and SW620 cells via real-time PCR after treatment with control (DMSO) or 5-aza-dC (**L**). Relative HINT2 protein intensity in cancer tissues relative to tumor stage (**M**) *P < 0.05, **P < 0.001 compared to SW620 cells. All data are representative of at least three independent experiments.

We selected 46 human CRC cases with para-cancer normal colorectal tissues. qRT-PCR and immunohistochemistry (IHC) analyses revealed that HINT2 was downregulated in CRC as compared to normal colorectal tissue (Figure 1A–1H), consistent with a previous study [10]. Multivariate analysis showed that HINT2 mRNA levels was downregulated in CRCs without lymph node metastasis than that in lymph node positive ones(*P* < 0.01; Figure 1I). IHC results in CRC liver and lymph node metastasis samples showed that HINT2 expression was low or absent in metastases (Figure 1C and 1F–1G). A semi-quantitative analysis revealed that HINT2 expression is progressively lost with increasing tumor stage (Figure 1M).

HINT2 expression was assessed in six cell lines with differing metastatic abilities (Caco-2, HCT-116, HT-29, SW480, SW48, SW620, and LoVo). HINT2 levels were lower in SW620 and LoVo lines derived from metastases than in the other four lines derived from primary tumors (Figure 1J–1K).

To determine whether DNA methylation was involved in transcriptional regulation of HINT2, LoVo and SW620 cells were treated with 5-aza-2′- deoxycytidine, a DNA demethylating agent. Treatment increased HINT2 levels in Lovo (3.4-fold) and SW620 (2.7-fold) cells (Figure 1L). These results suggested that DNA methylation might be involved in HINT2 downregulation.

### HINT2 downregulation promotes CRC cell migration and invasion

**Figure 2:**
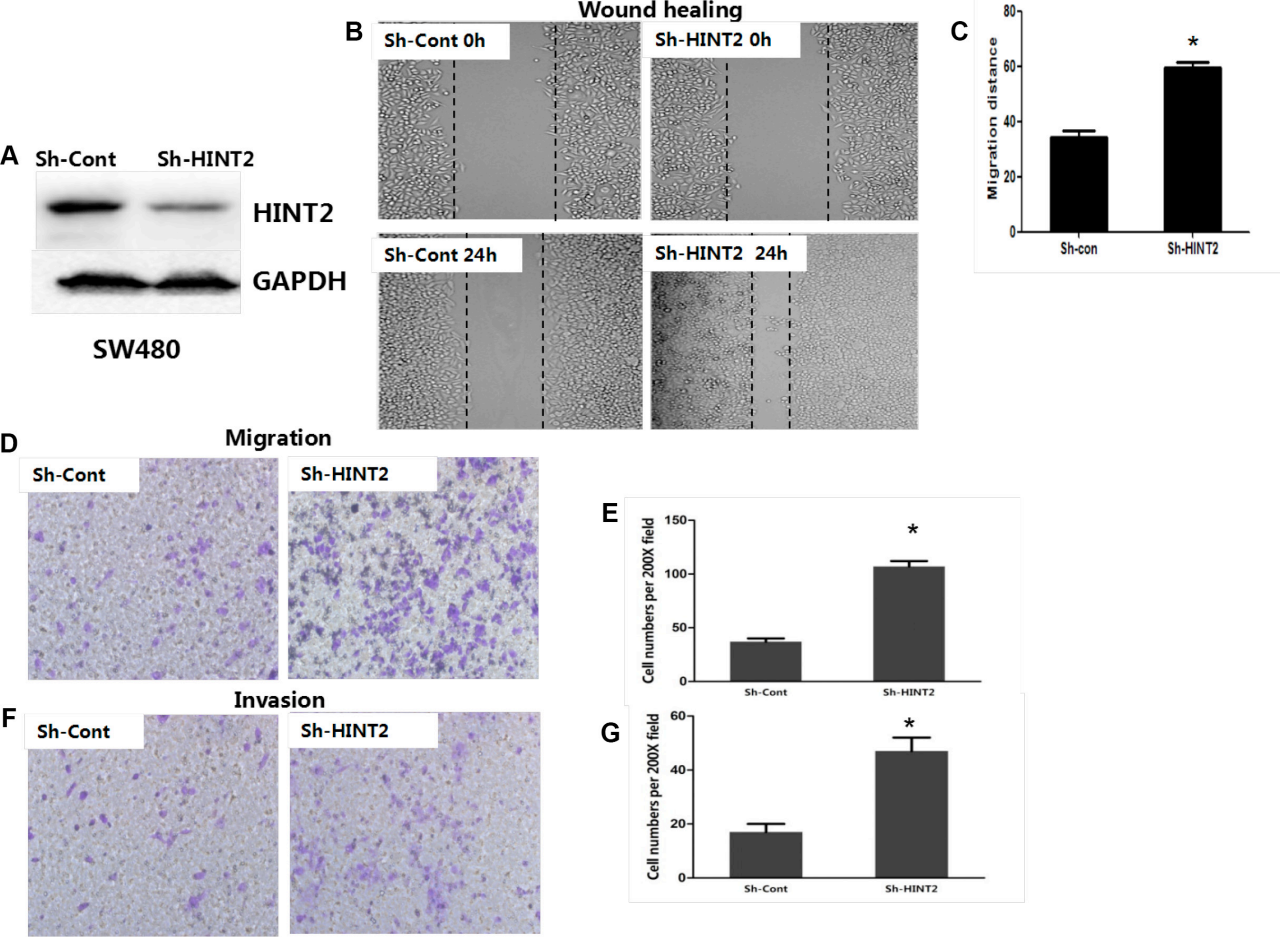
HINT2 downregulation is positively associated with CRC cell metastasis and invasion HINT2 knockdown in SW480 cells was confirmed by western blotting (**A**). Wound healing showing SW480 cell migration (**B** and **C**) after 24-h incubation. Cells were photographed under a phase contrast microscope. Transwell assay showing SW480 cell migration (**D** and **E**) after 48-h incubation (×200 magnification). Results were plotted as the average number of migrated cells from six random microscope fields. Transwell assay showing SW480 cell invasion (**F**) and qualification (**G**) after 48-h incubation (×200 magnification). Results were plotted as the average number of invading cells from six random microscope fields. All data are representative of at least three independent experiments. **P < 0.01, ***P < 0.001 compared to controls.

**Figure 3:**
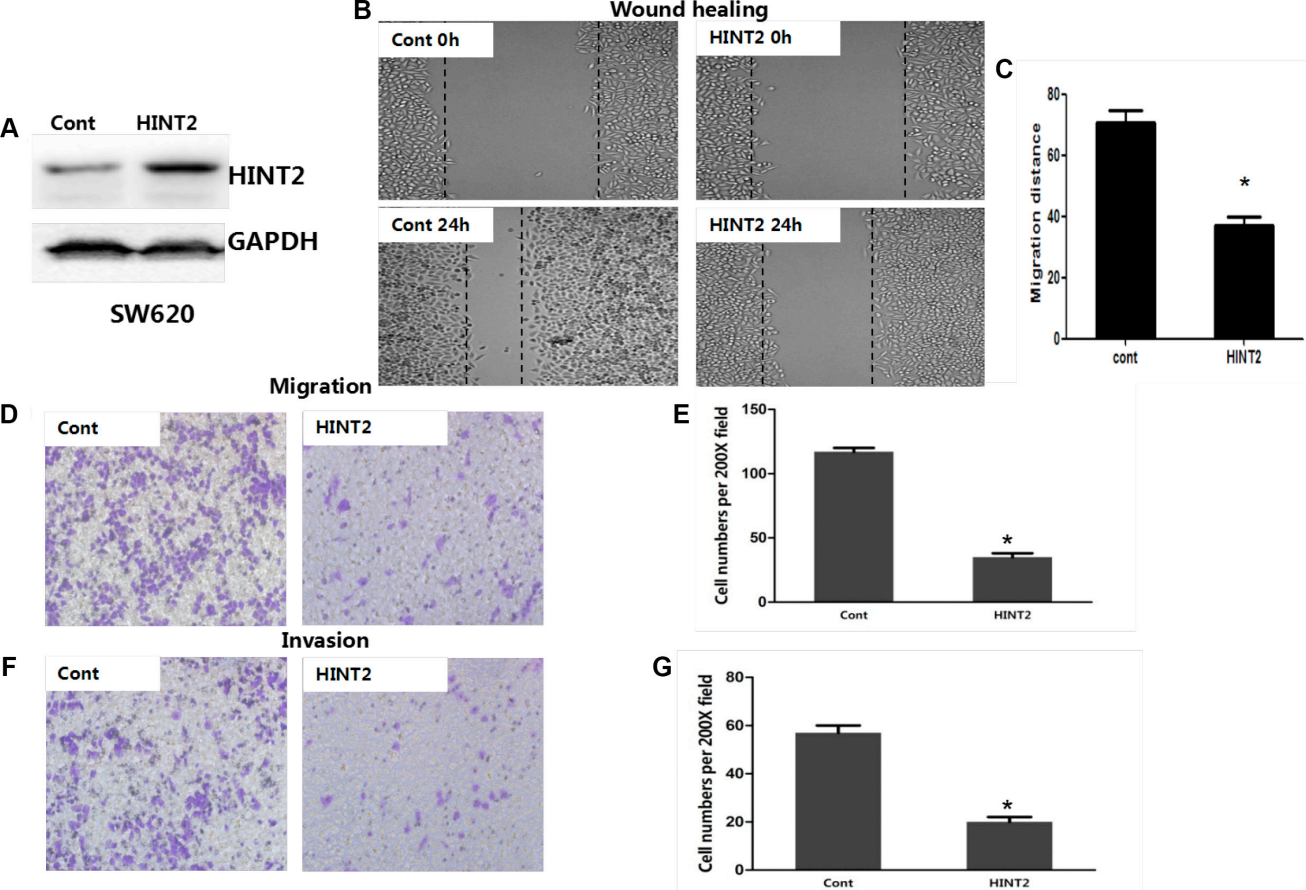
HINT2 overexpression is negatively associated with CRC cell migration and invasion HINT2 overexpression in SW620 cells was confirmed by western blotting (**A**). Wound healing assay showing SW620 cell migration (**B** and **C**) after 24-h incubation. Cells were photographed under a phase contrast microscope. Transwell assay showing SW620 cell migration (**D** and **E**) (×200 magnification) after 48-h incubation. Results were plotted as the average number of migrated cells from six random microscope fields. Transwell assay showing SW620 cell invasion (**F**) and qualification (**G**) (×200 magnification) after 48-h incubation. Results were plotted as the average number of invading cells from six random microscope fields. All data are representative of at least three independent experiments. **P < 0.01, ***P < 0.001 compared to controls.

HINT2 was knocked down in SW480 cells via shRNA. HINT2 expression changes were confirmed by western blotting (Figure 2A). To investigate whether EMT induction by HINT2 contributed to CRC cell migration and invasion, we first performed transwell migration assays in control and SW480 stable HINT2 knockdown cells. Cell migration was increased in knockdown cells (*P* < 0.01) (Figure 2D–2E). A wound-healing assay confirmed that HINT2 knockdown increased cell motility (*P* < 0.01) (Figure 2B–2C). A quantitative *in vitro* Matrigel invasion assay showed that HINT2 knockdown increased SW480 cell invasion (*P* < 0.01; Figure 2F–2G). We also transfected HINT2 into SW620 cells and confirmed its overexpression (Figure 3A). Using the same migration and invasion assays described above, we found that HINT2 overexpression decreased SW620 cell migration and invasion (Figure 3B–3F). Overall, these results support the hypothesis that HINT2 downregulation promotes CRC cell migration and invasion.

### HINT2 induces EMT

**Figure 4:**
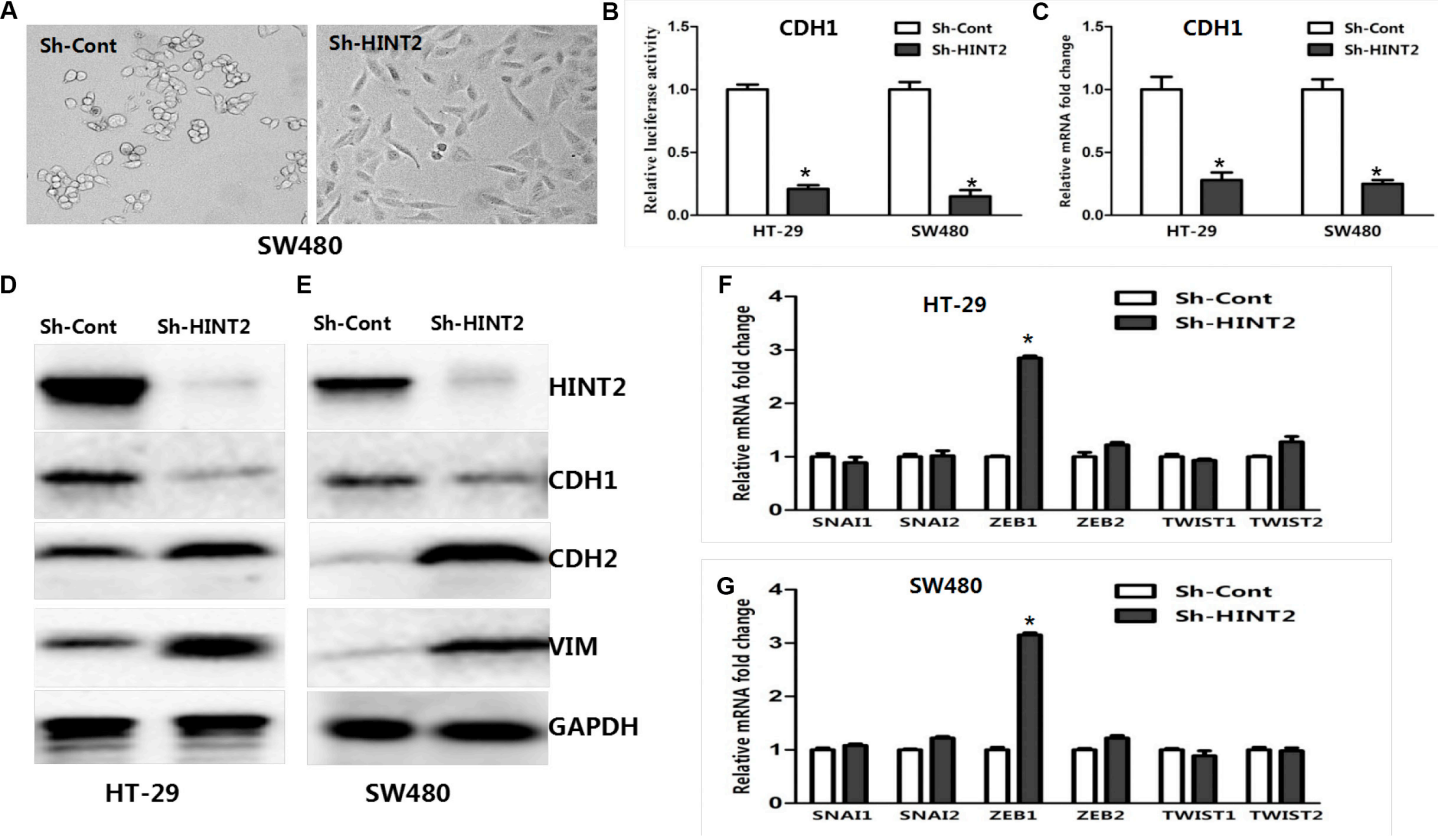
HINT2 downregulation induced EMT in CRC cells SW480 cells with and without HINT2 downregulation, photographed under a phase contrast microscope (**A**). A mesenchymal phenotype was observed in HINT2 knockdown cells. HT-29 and SW480 HINT2 knockdown cells were transfected with the CDH1 promoter vector, pGL-CDH1, and relative luciferase activation was detected (**B**). Data from control cells were set at 100%. RT-PCR analysis of EMT transcription factor, CDH1, in HT-29 and SW480 HINT2 knockdown cells compared with controls (**C**). Data from control cells were set at 100%. HINT2 and several EMT markers were detected by western blotting in HT-29 (**D**) and SW480 (**E**). HINT2-downregulated and control cells. HINT2-downregulated cells had increased CDH2 and vimentin expression and decreased CDH1 expression. GAPDH was used as the loading control. RT-PCR analysis of EMT transcription factors, such as SNAI1, SNAI2, ZEB1, ZEB2, TWIST1, and TWIST2 in HT-29 (**F**) and SW480 (**G**). HINT2-downregulated and control cells. Data from control cells were set at 100%. *P < 0.05, ***P < 0.001 compared to controls. All experiments are detected and analyzed in triplicates.

HINT2-downregulated SW480 cells changed from tightly packed to disseminated and diffusely organized (Figure 4A). To determine whether EMT molecular alterations occurred in these cells, expression of the epithelial marker E-cadherin (CDH1), and the mesenchymal markers, vimentin and N-cadherin (CDH2), were measured via western blotting. CDH1 was decreased while CDH2 and vimentin were increased in HINT2 knockdown SW480 and HT-29 cells as compared to controls (Figure 4D–4E).

To investigate the role of HINT2 in regulating CDH1 transcription, a luciferase reporter assay was performed. HT-29 and SW480 cells were transfected with pGL3/CDH1 along with a *Renilla* reporter plasmid. CDH1 luciferase activity was decreased (*P* < 0.01) in HINT2 knockdown cells compared with controls (Figure 4B). RT-PCR confirmed these results (Figure 4C). Taken together, these data imply that HINT2 induces an anti-EMT gene expression profile in CRC cell lines.

### Molecular markers confirm the essential role of ZEB1 in HINT2-induced EMT through HIF-2α

*SNAI1*, *SNAIL2*, *TWIST1*, *TWIST2*, *ZEB1*, and *ZEB2* mRNA levels were examined in HT-29 and SW480 cells. ZEB1 expression was higher in HINT2 knockdown cells than in controls (*P* < 0.05) (Figure 4F–4G), demonstrating that HINT2 expression in CRC cells correlates negatively with ZEB1 and positively with CDH1.

**Figure 5:**
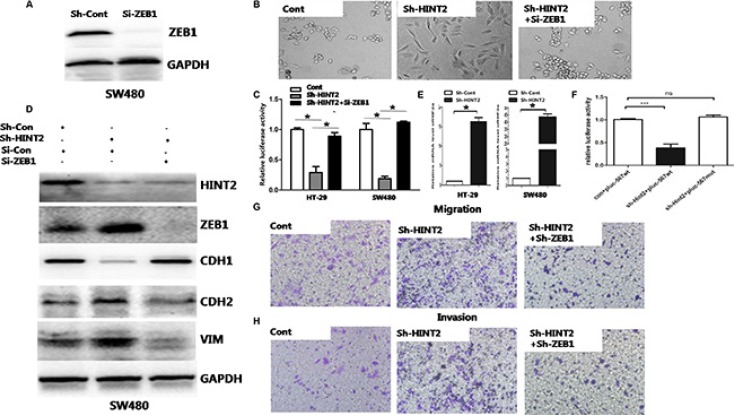
Molecular markers confirm the essential role of ZEB1 in HINT2-induced EMT through HIF-2α siRNA-mediated ZEB1 knockdown in SW480 HINT2-downregulated cell lines was confirmed by western blotting (**A**) ZEB1 inhibition rescues the mesenchymal phenotypic change (**B**) and CDH1 transcriptional inhibition (**C**) caused by HINT2 downregulation. HINT2 knockdown or control SW480 and HT-29 cells were transfected si-ZEB1 or si-Con (control), then transfected with the CDH1 promoter vector, pGL-CDH1, and relative luciferase activation was detected. Data from control cells were set at 100%. Western blot analysis of HINT2, ZEB1, and EMT markers in SW480 control, HINT2-knockdown, and HINT2/ZEB1 double knockdown cells (**D**) HINT2 and ZEB1 double knockdown nearly rescued the effect of HINT2 silencing alone on EMT. Real-time PCR analysis of HIF-2α in HT-29 and SW480 control and HINT2-knockdown cells (**E**) HINT2 knockdown enhanced HIF-2α expression. Activation of plu567 or pluc567-mut in SW480 cells with or without HINT2 knockdown (*n* = 3 replicate experiments) (**F**) Transwell assay showing SW480 cell migration (**G**) and invasion (**H**) after 48-h incubation (×200 magnification). Results were plotted as the average number of migrated cells from six random microscope fields. *P < 0.05, ***P < 0.001 compared to controls.

ZEB1 promotes migration and invasion by inducing EMT in colon cancer [9, 10]. To determine whether the negative correlation between HINT2 and CDH1 is mediated by ZEB1, we performed a luciferase reporter assay using human pGL3/CDH1. Stable HINT2 knockdown or control SW480 and HT-29 cells were transfected with control or ZEB1 siRNA, then transfected with the CDH1 promoter vector pGL-CDH1, and relative luciferase activity was detected. HINT2 downregulation repressed CDH1 transcription. When both ZEB1 and HINT2 were downregulated, there was no change in relative luciferase activity (Figure 5C).

Transwell assays with or without Matrigel showed that HINT2 and ZEB1 double knockdown largely prevented migration and invasion induced by HINT2 downregulation alone (*P* < 0.01) (Figure 5G–5H). HINT2 and ZEB1 double knockdown also blocked EMT promotion caused by HINT2 downregulation alone (Figure 5B and 5D).

A previous study showed that HINT2 knockout mice had increased levels of HIF-2α and reactive oxygen species [20]. We performed a qRT-PCR assay to measure HIF-2α expression in HT-29 and SW480 cells. We found that the HIF-2α expression increased in HT-29 and SW480 cells after down-regulation of Hint2, which is consistence with previously report (Figure 5E). HIF-1α and HIF-2α share potential binding sequences in the ZEB1 promoter. HIF-1α reportedly binds exclusively to hypoxia response element (HRE) 3 (634–629nt) [21]. We mutated HRE1 (521–516 nt) and HRE2 (529–524 nt) and found no changes in ZEB1 relative luciferase activity upon changes in HINT2 expression (Figure 5F).

### HINT2 downregulation promotes CRC metastasis *in vivo*

**Figure 6:**
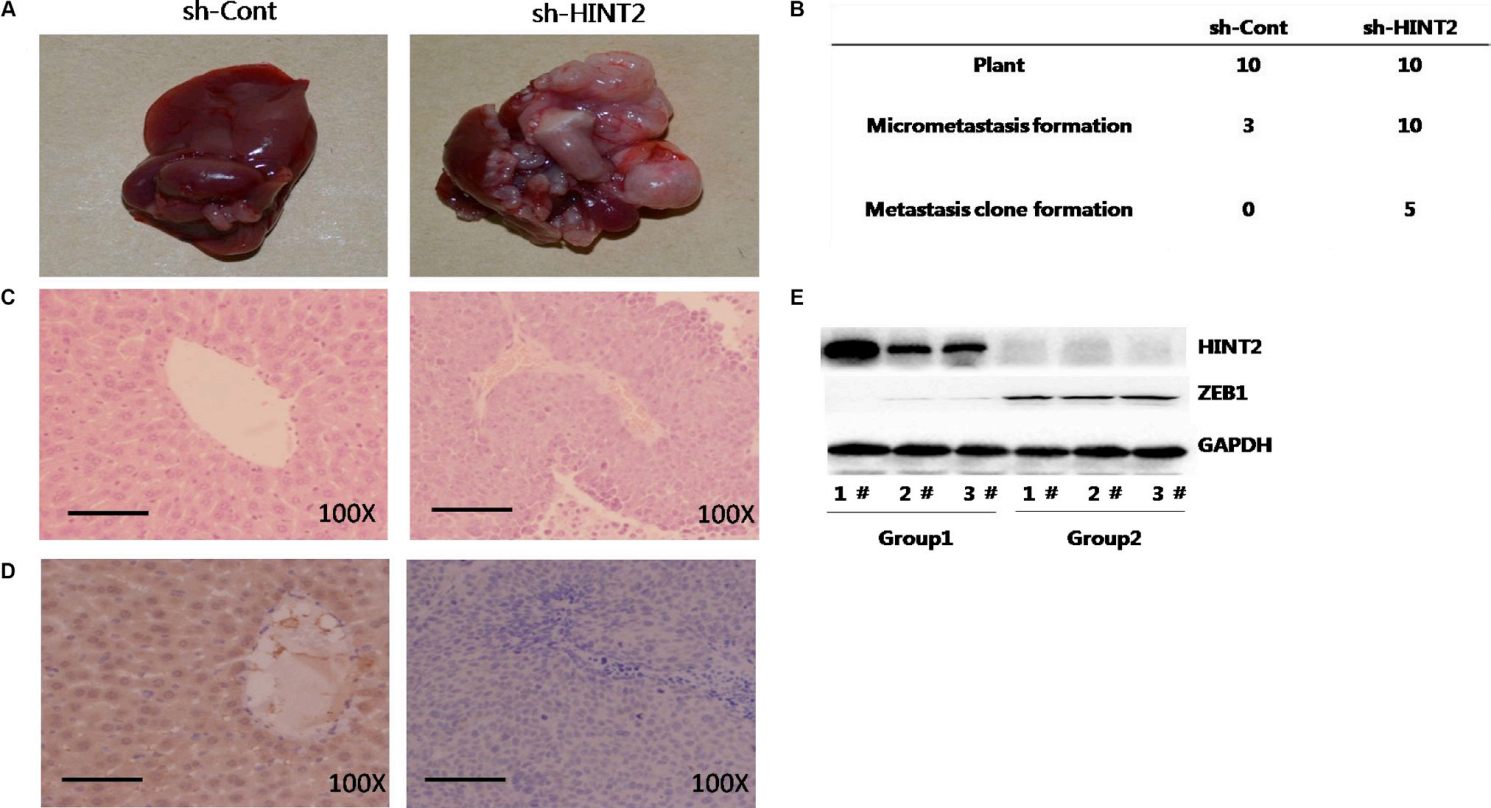
HINT2 downregulation promotes CRC metastasis *in vivo* SW480 cells with and without HINT2 knockdown were injected intrasplenically into female BALB/c NOD mice, and mice were killed six weeks later. Tumor islets were observed in livers via H&E staining. Typical views of liver presenting macroscopic metastases at week six (**A**) Left panel: SW480 without HINT2 knockdown; right panel: SW480 with HINT2 knockdown. Numbers of liver metastases and micrometastases after intrasplenic injection (**B**). H&E (**C**) and HINT2 (**D**) staining in mouse metastatic liver tumor tissues. Western blot analysis of HINT2 and ZEB1 in liver metastases from control (group 1) and HINT2-downregulated (group 2) SW480 cell-injected mice (**E**) All experiments were performed in triplicate.

Control and HINT2 knockdown SW480 cells were injected into mice. Liver metastases were observed in five mice from the experimental group while no visible metastases formed in any control mice (Figure 6A–6B). Histologically, metastatic nests were found in all mice injected with HINT2 knockdown SW480 cells, and in three mice from the control group. Metastatic foci in the knockdown group were larger than those in the control group as measured by HE staining (Figure 4C). IHC results showed that HINT2 was absent in the metastatic nests and tiny foci of the knockdown group, while HINT2 staining was strong in the control group (Figure 4D). Together, these results indicate that ZEB1 mediates HINT2-dependent EMT regulation through HIF-2α.

## DISCUSSION

Metastasis is the most significant contributor to cancer death, and the EMT is a fundamental event in this process [22–24]. Silencing EMT-related genes is an attractive therapeutic approach for improving treatment outcomes [25–27]. The present study found that HINT2 downregulation promotes CRC cell migration and invasion by enhancing the ZEB1-mediated EMT response through HIF-2α.

HINT2 expression is generally downregulated in human hepatocellular carcinoma cells and HINT2 appears to sensitize these cells to apoptosis [10]. We hypothesized that HINT2 downregulation also promotes CRC progression. We confirmed that HINT2 expression was lower in CRC tissues than in normal colon mucosa, especially during metastasis, and that HINT2 abundance was inversely correlated with CRC tumor stage. Two cell lines derived from metastases exhibited lower HINT2 levels, suggesting that HINT2 loss promotes this process. We observed that HINT2 knockdown induced cell infiltration through transwell membranes, while HINT2 overexpression inhibited this process. In mice injected with CRC cells, HINT2 downregulation induced the formation of more and larger colonies in the liver, indicating that reduced HINT2 promoted CRC metastasis by promoting cell migration and invasion.

The EMT is important for tumor cells to acquire migratory and invasive properties [28, 29], and was recently implicated in several types of invasive human malignancies [30–33], including CRC [34]. Hence, EMT and its markers, including CDH1, are useful diagnostic biomarkers or therapeutic targets in CRC. In the present study, HINT2 downregulation enhanced CDH2 and vimentin expression and suppressed CDH1 in CRC cells. The outcome of a luciferase reporter assay using the CDH1 promoter supports transcriptional regulation as the major mechanism underlying HINT2-dependent EMT marker expression changes. Therefore, our work provides evidence that HINT2 downregulation may enhance CRC cell migration and invasion by inducing EMT.

Several cell signaling pathways, including transforming growth factor-β [35], Wnt [36], and growth factor receptor signaling cascades [37–39], regulate EMT and the progression of various cancers, including CRC. These signaling axes induce EMT by repressing CDH1, an EMT epithelial marker. To investigate the mechanisms by which HINT2 regulates the EMT in CRC, we measured six upstream transcription factors (SNAI1, SNAIL2, TWIST1, TWIST2, ZEB1, and ZEB2) that suppress CDH1 transcription. HINT2 knockdown increased ZEB1 expression specifically, suggesting that HINT2 may regulate the EMT by downregulating ZEB1.

Given the pivotal role of ZEB1 in downregulating CDH1 and inducing the loss of cell polarity, ZEB1 drives the EMT and cancer progression [40, 41]. We assessed the importance of the interaction between HINT2 and ZEB1 in regulating EMT in CRC cells. We knocked down ZEB1 in stable HINT2-silenced HT-29 and SW480 cells and then examined cell morphology and EMT marker expression. The effects of HINT2 on the EMT response were rescued by ZEB1 knockdown. The luciferase assay further demonstrated that HINT2 downregulation induced CDH1 transcription in a ZEB1-dependent manner in CRC cells.

HIF-2α is an important angiogenic factor with prognostic value, and high HIF-2α expression in CRC promotes tumor progression [42]. We confirmed the results of a previous study showing that HINT2 knockout mice had increased HIF-2α levels. To evaluate whether ZEB1 mediated HINT2-dependent regulation of the EMT program through HIF-2α, the promoter sequences of ZEB1 were analyzed using bioinformatics methods. As transcription factors, HIF-1α and HIF-2α regulate downstream genes by binding HRE sites in their promoter regions. We found three potential HRE sites in the proximal promoter (approximately 1000 nt upstream; the start codon ATG defined as 0) of the ZEB1 gene: HRE-1 at 517–521 nt; HRE-2 at 525–529 nt; and HRE-3 at 630–634 nt. HIF-1α directly regulated ZEB1 expression through HRE-3. Mutation of both HRE1 and HRE2 revealed no changes in ZEB1 relative luciferase activity after altering HINT2 expression, and demonstrated that HINT2 downregulation leads direct HIF-2α activation of ZEB1. This is the first evidence that HINT2 downregulation promotes CRC metastasis through HIF-2α-mediated regulation of ZEB1 expression. HINT2 might be a useful clinical biomarker, indicating CRC progression and metastasis risk.

## MATERIALS AND METHODS

### Cell lines and reagents

Dulbecco's modified Eagle's medium (DMEM), Leibovitz Medium L-15 (L-15 medium), McCoy's 5a medium, DMEM/F12, fetal bovine serum (FBS), and 0.25% trypsin were purchased from Hyclone (GE Healthcare Life Sciences, Logan, UT, USA). SW480 and SW620 cells were cultured in L-15 medium supplemented with 10% fetal bovine serum (FBS). HT-29 and HCT-116 cells were cultured in McCoy's 5a complete medium. LoVo cells were cultured in DMEM/F12 supplemented with 10% FBS. Caco-2 cells were cultured in DMEM supplemented with 10% FBS. All cells were purchased from the Type Culture Collection cell bank (Chinese Academy of Sciences, Beijing, P.R. China) and maintained in a 5% CO_2_ humidified atmosphere at 37°C.

Antibodies against CDH1 (E-cardherin), CDH2 (N-cardherin), and VIM (Vimentin) were from Cell Signaling Technology, Inc. (USA). Antibodies against HINT2 and GAPDH were from Santa Cruz Biotechnology (Santa Cruz, CA, USA). Lentivirus plasmids were purchased from Addgene (Cambridge, MA, USA); other chemical reagents were purchased from Takara (Dalian, P.R. China) or Promoter Company (Wuhan, P.R. China).

### Plasmid generation, transfection, and gene silencing

Full-length human HINT2 was isolated, and cDNA was sub-cloned into the pLVX-IRES-ZsGreen1 plasmid using the following primers: F: 5′-CGG AATTCATGGCGGCAGCCGTGGTGCTGG-3′and R: 5′ -GGACTAGTTCAACCTGGAGGCCACTGGAGC-3′. Li-pofectamine^®^2000 reagent and Opti-MEM (Invitrogen) were used for cell transfection. After transfection, cell were incubated at 37°C/5% CO_2_ for 24–48 h prior to transgene expression testing. The short hairpin RNA (sh)-HINT2-sense (5′-GGUUGAACCUGCCAACUGAdTdT-3′) and sh-HINT2-antisense (5′-UCAGUUGGCAGGUUCAACC dTdT-3′) sequences were purchased from RiboBio (Guangzhou, P.R. China). The ZEB1 short-interfering RNA (siRNA) target sequence was, 5′-GACCAGAACAGUGU UCCAUGUUUAA-3′. Lentiviruses were produced by co-transfecting HEK293T cells with lentiviral packaging plasmids (2.2 μg of pCMV-dR8.91, 0.25 ng of VSV-G/pMD2G, and 2.5 μg of Hairpin-pLKO.1). Supernatants were applied to CRC cells at an equivalent titer. After 72 h, infected cells were selected with puromycin for 5 d, and at least 500 resistant clones from each group were pooled.

### Human colorectal cancer samples

Human CRC specimens (including one with liver metastatic tissue) were obtained from 46 CRC patients treated in the Department of Oncological Surgery, Fujian Provincial Hospital, Fuzhou, China. All patients had consented to tissue collection for research at the time of surgery. All specimens were diagnosed as CRC by the Department of Pathology, Fujian Provincial Hospital. No patient had received chemotherapy, immunotherapy or radiotherapy prior to specimen collection. Tissue samples were divided into two portions: the first portion was frozen at −80°C for mRNA isolation and the second was used for pathological examination. This research was approved by the Ethics Committee of Fujian Medical University.

### Immunohistochemistry (IHC)

Paraffin-embedded tissue sections were dewaxed and heated in a pressure cooker. Endogenous peroxidase activity was blocked using H_2_O_2_. Sections were incubated with normal serum buffer for 30 min and then with primary antibodies (diluted 1:100) for 1 h at room temperature. Sections were then incubated with horseradish peroxidase-conjugated secondary antibodies before a DAB kit (BosterBio, Wuhan) was used to develop the color reaction. Sections were counter-stained with hematoxylin and eosin (HE), and dehydrated using xylene and alcohol before mounting. Images were acquired with a Leica2000 fluorescence microscope. IHC staining intensity was measured using Image-Pro Plus software (Media Cybernetics, Rockville, MD, USA) in three randomly selected high-power (×400) fields per section.

### Pharmacological DNA demethylation with 5-aza-2′-deoxycytidine

Lovo and SW620 Cells were treated for 72 h with 5mM 5-aza-2′-deoxycytidine (5-aza-dC) (Sigma, St Louis, MO, USA), a well-used methyltransferase inhibitor. 5-aza-dC was replenished every 24 h. An equivalent concentration of the vehicle (DMSO) was used as the control.

### Luciferase reporter assay

To examine transcriptional regulation of CDH1 promoters, the CDH1 promoter sequence was amplified from normal human genomic DNA and subcloned into a pGL3-basic luciferase reporter vector (Promega) to generate the CDH1 promoter/firefly luciferase reporter construct, pGL3-CDH1.

Production of wildtype and mutant reporter constructs was performed as previously described [43]. A 567 bp ZEB1 promoter fragment containing HRE (hypoxia response element)-1, HRE-2 and HRE-3 was cloned into the pGL3 vector to generate the wildtype reporter construct, pluc-567wt. Mutations at both HRE-1 and HRE-2 motifs (pluc-567-mut) were introduced into the pluc-567 luciferase construct using the QuikChange Site-Directed Mutagenesis Kit (Stratagene, La Jalla, CA, USA). Both constructs were verified by sequencing. SW480 cells with and without sh-HINT2 were transfected with pluc-567-wt or pluc-567-mut, and Renilla luciferase.

48 h after transfection, luciferase activities were measured using a Dual-Luciferase Reporter Assay System (Promega), and results were normalized to *Renilla* luciferase activity. Firefly luciferase activity in cells transfected with sh-HINT2 was represented as the percentage of activity relative to that of cells transfected with control shRNA. All experiments were repeated at least in triplicate.

### Quantitative real-time PCR (qRT-PCR)

Total RNA was extracted from cells using TRizol (Invitrogen) according to the manufacturer's instructions. cDNA was synthesized using a PrimeScript^®^ RT reagent kit (Takara Biotechnology). mRNA levels were detected in triplicate with SYBR^®^ Premix Ex Taq (Takara). Transcript levels are expressed relative to levels observed in experimental or control cells and tissues, which was set to 1.0. Relative gene expression was calculated using the comparative threshold value (ΔΔCt) method. Primers used for qRT-PCR analysis were as follows: ZEB1-F (5′-GCCAATAAGCAAACGATTCTG-3′), ZEB1-R (5′-TT TGGCTGGATCACTTTCAAG-3′), ZEB2-F (5′-CGGTG CAAGAGGCGCAAACA-3′), ZEB2-R (5′-GGAGGA CTCATGGTTGGGCA-3′), Snail1-F (5′-CACTATGCCG CGCTCTTTC-3′), Snail1-R (5′-GGTCGTAGGGCTGC TGGAA-3′), Snail2-F (5′-AAACTACAGCGAACTGGA CACA-3′), Snail2-R (5′-GCCCCAAAGATGAGGAGT ATC-3′), Twist1-F (5′-AGTCCGCAGTCTTACGAGGA -3′), Twist1-R (5′-GCCAGCTTGAGGGTCTGAAT-3′), Twist2-F (5′-CAAGCTGAGCAAGATCCAGAC3′), Twist 2-R (5′-GGTCATCTTATTGTCCATCTCG-3′), ZEB1-F (5′-GATGATGAATGCGAGTCAGATGC-3′), ZEB1-R (5′-ACAGCAGTGTCTTGTTGTTGT-3′), ZEB2-F (5′-GT GACAAGACATTCCAGAAAAGCAG-3′), ZEB2-R (5′ -GAGTGAAGCCTTGAGTGCTC-3′), GAPDH-F (5′-GG AGCGAGATCCCTCCAAAAT-3′), GAPDH-R (5′-GGC TGTTGTCATACTTCTCATGG-3′), CDH1-F (5′-TAC ACTGCCCAGGAGCCAGA-3′), CDH1-R (5′-TGGCAC CAGTGTCCGGATTA-3′). HIF2α-F (5′-GCTCTCCACG GCCTGATA-3′), HIF2α-R (TTGTCACAC-CTATGGCAT ATCACC-3′). Gene transcript levels were normalized to GAPDH.

### Western blotting

Total proteins were extracted from cells or tissue. Equal amounts of protein were resolved using sodium dodecyl sulfate–polyacrylamide gel electrophoresis (SDS-PAGE) (12.5%), transferred onto a nitrocellulose membrane, and then blocked with 5% non-fat dry milk in phosphate-buffered saline (PBS)–Tween 20 containing 1% (wt/vol) bovine serum albumin (BSA). After blocking, membranes were incubated with polyclonal antibodies against HINT2, CDH1, CDH2, Vimentin and GAPDH, followed by immunoglobulin G (IgG) secondary antibody at 4°C overnight. Immunoreactive proteins were then detected using an enhanced chemiluminescence detection system (Amersham, GE Healthcare Bio-Sciences, Pittsburgh, PA, USA). Equal protein loading was confirmed using GAPDH. The experiment was performed three times in triplicate.

### Transwell migration and invasion assays

Cells (5 × 10^5^/mL, 200 μL/well) in serum-free medium were loaded into the upper chambers of Transwell inserts (BD Biosciences, San Jose, CA, USA). The lower chambers were filled with medium containing serum. After 48-h incubation, cells on the upper surfaces of the inserts were removed, and cells on the lower surface of the membrane were fixed with 100% methanol and stained with 0.1% crystal violet. To minimize bias, cells from at least six randomly selected fields were counted and averaged under ×200 magnification, and images were captured using an inverted light microscope. Each study was conducted in triplicate in three independent experiments. For the invasion assay, 5 × 10^6^/mL (200 μL/well) cells were loaded into the top of a 24-well Matrigel-coated insert (8-μm pores; BD Biosciences) according to the manufacturer's protocol. After 48 h, non-invading cells were removed using cotton swabs. Cells that had migrated to the bottom of the membrane were fixed with 100% methanol and stained with 0.1% crystal violet. Invading cells were counted in the same manner as in migration assays.

### Wound-healing assay

Cells were seeded in 6-well plates and incubated until 90% confluent. The wound was generated by scratching the monolayer surface with a 200-μL pipette tip, and cells were then washed three times with PBS to remove cell debris. Images were obtained by microscopy at 0 and 24 h, and distances covered by migrated cells were quantified.

### Animal models

A liver metastasis assay was used to determine the effects of HINT2 on metastasis. Our study was performed in accordance with institutional guidelines, and was approved by the Animal Experimentation Committee of the Central Institute for Experimental Animals, Fujian Medical University. We bred female BALB/c NOD mice to 4–5 weeks of age. Colon cancer cells were harvested with 0.25% trypsin-EDTA solution, washed, and suspended in serum-free medium at 2 × 10^5^ cells/mL. Experimental liver metastases were generated via intrasplenic injection of 1 × 10^5^ cancer cells (50 μL of cell suspension) and splenectomy. Mice were sacrificed six weeks later and liver metastases enumerated immediately without fixation. Numbers of metastatic liver clones in each liver, and small foci were compared in sh-HINT2 group and sh-control group.

### Statistical analysis

Each study was repeated at least three times. Data are presented as the mean ± SD. All statistical analyses were conducted with SPSS software version 13.0 (SPSS Inc., Chicago, IL, USA). Between-group and among-group comparisons were conducted using Student's *t*-tests and analysis of variance (ANOVA), respectively. *P* < 0.05 was considered statistically significant.

## References

[1] Lee WS, Yun SH, Chun HK, Lee WY, Yun HR, Kim J, Kim K, Shim YM (2007). Pulmonary resection for metastases from colorectal cancer: prognostic factors and survival. Int J Colorectal Dis.

[2] Van Cutsem E, Nordlinger B, Adam R, Kohne CH, Pozzo C, Poston G, Ychou M, Rougier P (2006). Towards a pan-European consensus on the treatment of patients with colorectal liver metastases. Eur J Cancer.

[3] Tamandl D, Gruenberger B, Klinger M, Herberger B, Kaczirek K, Fleischmann E, Gruenberger T (2010). Liver resection remains a safe procedure after neoadjuvant chemotherapy including bevacizumab: a case-controlled study. Ann Surg.

[4] Huang XF, Chen JZ (2009). Obesity, the PI3K/Akt signal pathway and colon cancer. Obes Rev.

[5] Martin J, Magnino F, Schmidt K, Piguet AC, Lee JS, Semela D, St-Pierre MV, Ziemiecki A, Cassio D, Brenner C, Thorgeirsson SS, Dufour JF (2006). Hint2, a mitochondrial apoptotic sensitizer down-regulated in hepatocellular carcinoma. Gastroenterology.

[6] Martin J, Maurhofer O, Bellance N, Benard G, Graber F, Hahn D, Galinier A, Hora C, Gupta A, Ferrand G, Hoppeler H, Rossignol R, Dufour JF (2013). Disruption of the histidine triad nucleotide-binding hint2 gene in mice affects glycemic control and mitochondrial function. Hepatology.

[7] Amoedo ND, Rodrigues MF, Rumjanek FD (2014). Mitochondria: are mitochondria accessory to metastasis?. Int J Biochem Cell Biol.

[8] Stram AR, Payne RM (2016). Post-translational modifications in mitochondria: protein signaling in the powerhouse. Cell Mol Life Sci.

[9] Peiris-Pages M, Martinez-Outschoorn UE, Pestell RG, Sotgia F, Lisanti MP (2016). Cancer stem cell metabolism. Breast Cancer Res.

[10] Martin J, St-Pierre MV, Dufour JF (2011). Hit proteins, mitochondria and cancer. Biochim Biophys Acta.

[11] Tsai JH, Yang J (2013). Epithelial-mesenchymal plasticity in carcinoma metastasis. Genes Dev.

[12] Altavilla G, Marchetti M, Padovan P, Marcato E, Onnis A (1996). Predictive value of proliferative cellular nuclear antigen (PCNA) and Ki-67 antigen in advanced stage serous papilliferous ovarian cancer. Eur J Gynaecol Oncol.

[13] Jing Y, Cui D, Guo W, Jiang J, Jiang B, Lu Y, Zhao W, Wang X, Jiang Q, Han B, Xia S (2014). Activated androgen receptor promotes bladder cancer metastasis via Slug mediated epithelial-mesenchymal transition. Cancer Lett.

[14] Wang G, Yang X, Li C, Cao X, Luo X, Hu J (2014). PIK3R3 induces epithelial-to-mesenchymal transition and promotes metastasis in colorectal cancer. Mol Cancer Ther.

[15] Xie D, Gore C, Liu J, Pong RC, Mason R, Hao G, Long M, Kabbani W, Yu L, Zhang H, Chen H, Sun X, Boothman DA (2010). Role of DAB2IP in modulating epithelial-to-mesenchymal transition and prostate cancer metastasis. Proc Natl Acad Sci USA.

[16] Zhang WG, Li CF, Liu M, Chen XF, Shuai K, Kong X, Lv L, Mei ZC (2016). Aquaporin 9 is down-regulated in hepatocellular carcinoma and its over-expression suppresses hepatoma cell invasion through inhibiting epithelial-to-mesenchymal transition. Cancer Lett.

[17] Rojas-Puentes L, Cardona AF, Carranza H, Vargas C, Jaramillo LF, Zea D, Cetina L, Wills B, Ruiz-Garcia E, Arrieta O (2016). Epithelial-mesenchymal transition, proliferation, and angiogenesis in locally advanced cervical cancer treated with chemoradiotherapy. Cancer Med.

[18] Spaderna S, Schmalhofer O, Wahlbuhl M, Dimmler A, Bauer K, Sultan A, Hlubek F, Jung A, Strand D, Eger A, Kirchner T, Behrens J, Brabletz T (2008). The transcriptional repressor ZEB1 promotes metastasis and loss of cell polarity in cancer. Cancer Res.

[19] Zhang GJ, Zhou T, Tian HP, Liu ZL, Xia SS (2013). High expression of ZEB1 correlates with liver metastasis and poor prognosis in colorectal cancer. Oncol Lett.

[20] Anderson Kristin A., Wang Dongning, Matthew D (2013). Hirschey.HINT2 and Fatty Liver Disease: Mitochondrial Protein Hyperacetylation Gives a Hint?. Hepatology.

[21] Zhang Wenjing, Shi Xinpeng, Peng Ying, Wu Meiyan, Zhang Pei, Xie Ruyi, Wu Yao, Yan Qingqing, Liu Side, Wang Jide (2015). HIF-1α Promotes Epithelial-Mesenchymal Transition and Metastasis through Direct Regulation of ZEB1 in Colorectal Cancer. PLoS One.

[22] Kalluri R, Weinberg RA (2009). The basics of epithelial-mesenchymal transition. J Clin Invest.

[23] Cao H, Xu E, Liu H, Wan L, Lai M (2015). Epithelial-mesenchymal transition in colorectal cancer metastasis: A system review. Pathol Res Pract.

[24] Zhang P, Sun Y, Ma L (2015). ZEB1: at the crossroads of epithelial-mesenchymal transition, metastasis and therapy resistance. Cell Cycle.

[25] Masuda T, Hayashi N, Iguchi T, Ito S, Eguchi H, Mimori K (2016). Clinical and biological significance of circulating tumor cells in cancer. Mol Oncol.

[26] Dhamija S, Diederichs S (2016). From junk to master regulators of invasion: lncRNA functions in migration, EMT and metastasis. Int J Cancer.

[27] Smith BN, Bhowmick NA (2016). Role of EMT in Metastasis and Therapy Resistance. J Clin Med.

[28] Henry CE, Llamosas E, Djordjevic A, Hacker NF, Ford CE (2016). Migration and invasion is inhibited by silencing ROR1 and ROR2 in chemoresistant ovarian cancer. Oncogenesis.

[29] Dong XC, Jing LM, Wang WX, Gao YX (2016). Down-regulation of SIRT3 promotes ovarian carcinoma metastasis. Biochem Biophys Res Commun.

[30] Iwahashi S, Shimada M, Utsunomiya T, Imura S, Morine Y, Ikemoto T, Takasu C, Saito Y, Yamada S (2016). Epithelial-mesenchymal transition-related genes are linked to aggressive local recurrence of hepatocellular carcinoma after radiofrequency ablation. Cancer Lett.

[31] Guo Q, Qin W (2015). DKK3 blocked translocation of beta-catenin/EMT induced by hypoxia and improved gemcitabine therapeutic effect in pancreatic cancer Bxpc-3 cell. J Cell Mol Med.

[32] Gonzalez-Guerrico AM, Espinoza I, Schroeder B, Park CH, Kvp CM, Khurana A, Corominas-Faja B, Cuyas E, Alarcon T, Kleer C, Menendez JA, Lupu R (2016). Suppression of endogenous lipogenesis induces reversion of the malignant phenotype and normalized differentiation in breast cancer. Oncotarget.

[33] Sponziello M, Rosignolo F, Celano M, Maggisano V, Pecce V, De Rose RF, Lombardo GE, Durante C, Filetti S, Damante G, Russo D, Bulotta S (2016). Fibronectin-1 expression is increased in aggressive thyroid cancer and favors the migration and invasion of cancer cells. Mol Cell Endocrinol.

[34] Zhu QC, Gao RY, Wu W, Qin HL (2013). Epithelial-mesenchymal transition and its role in the pathogenesis of colorectal cancer. Asian Pac J Cancer Prev.

[35] Liu S, de Boeck M, van Dam H, Ten Dijke P (2016). Regulation of the TGF-beta pathway by deubiquitinases in cancer. Int J Biochem Cell Biol.

[36] Xu L, Zhang L, Hu C, Liang S, Fei X, Yan N, Zhang Y, Zhang F (2016). WNT pathway inhibitor pyrvinium pamoate inhibits the self-renewal and metastasis of breast cancer stem cells. Int J Oncol.

[37] Zaldumbide L, Erramuzpe A, Guarch R, Pulido R, Cortes JM, Lopez JI (2016). Snail heterogeneity in clear cell renal cell carcinoma. BMC Cancer.

[38] Kato T, Kameoka S, Kimura T, Nishikawa T, Kobayashi M (2002). C-erbB-2 and PCNA as prognostic indicators of long-term survival in breast cancer. Anticancer Res.

[39] Choudhary KS, Rohatgi N, Halldorsson S, Briem E, Gudjonsson T, Gudmundsson S, Rolfsson O (2016). EGFR Signal-Network Reconstruction Demonstrates Metabolic Crosstalk in EMT. PLoS Comput Biol.

[40] Voutsadakis IA (2016). Epithelial-Mesenchymal Transition (EMT) and Regulation of EMT Factors by Steroid Nuclear Receptors in Breast Cancer: A Review and in Silico Investigation. J Clin Med.

[41] Yoshida T, Song L, Bai Y, Kinose F, Li J, Ohaegbulam KC, Ohaegbulam KC, Munoz-Antonia T, Qu X, Eschrich S, Uramoto H, Tanaka F, Nasarre P (2016). ZEB1 Mediates Acquired Resistance to the Epidermal Growth Factor Receptor-Tyrosine Kinase Inhibitors in Non-Small Cell Lung Cancer. PLoS One.

[42] Alam Muhammad Wasi, Persson Camilla Ulrika, Reinbothe Susann, Kazi Julhash U., Rönnstrand Lars, Wigerup Caroline, Ditzel Henrik Jorn, Lykkesfeldt Anne E., Påhlman Sven, Jögil Annika (2016). HIF2α contributes to antiestrogen resistance via positive bilateral crosstalk with EGFR in breast cancer cells. Oncotarget.

[43] Zhang W, Wang J, Zou B, Sardet C, Li J, Lam CS, Ng L, Pang R, Hung IF, Tan V P, Jiang B, Wong BC (2011). Four and a half LIM protein 2 (FHL2) negatively regulates the transcription of E-cadherin through interaction with Snail1. Eur J Cancer.

